# Imbalanced shift of cytokine expression between T helper 1 and T helper 2 (Th1/Th2) in intestinal mucosa of patients with post-infectious irritable bowel syndrome

**DOI:** 10.1186/1471-230X-12-91

**Published:** 2012-07-20

**Authors:** Ji Chen, Yangde Zhang, Zhansheng Deng

**Affiliations:** 1 National Hepatobiliary & Enteric Surgery Research Center, Ministry of Health, Central South University, Changsha, 410008, China; 2 Digestive System Department, Baogang Hospital, the 3rd affiliated Hospital of Inner Mongolia Medical College, Baotou, 014010, China; 3 Department of Orthopedics, Xiangya Hospital, Central South University, Changsha, 410008, China

**Keywords:** Irritable bowel syndrome, Intestinal mucosa, Th1/Th2, Cytokine

## Abstract

**Background:**

Irritable bowel syndrome (IBS) is a common functional bowel disorder. The post-infectious IBS (PI-IBS) occurs in IBS patients with a history of intestinal infection preceding the onset of symptoms. However, the underlying cause of PI-IBS is not fully understood, and the purpose of this study was to investigate the immune regulatory mechanism of PI-IBS.

**Methods:**

Participants enrolled in this study were divided into three groups including PI-IBS patients (n = 20), IBS patients without a history of infection (non-PI-IBS, n = 18), and healthy controls (n = 20). The expression levels of the Th1-derived cytokines IFN-γ and IL-12, and the Th2-derived cytokines IL-4 and IL-10 in the mucosal specimens, and in the ascending colon, the descending colon, and the rectal segments were measured by RT-PCR and western blot.

**Results:**

The IFN-γ mRNA levels in the intestinal mucosa were significantly higher in the PI-IBS group than in the non-PI-IBS or control group (both P < 0.05), but there was no difference between the non-PI-IBS and control groups. A trend toward IFN-γ protein upregulation was found in the PI-IBS group, while the IL-12 and IL-4 mRNA and protein levels were not different between any groups. The IL-10 mRNA and protein levels in the PI-IBS group were both significantly lower than in the non-PI-IBS or control groups (P < 0.05, respectively), but there was no difference between the non-PI-IBS and control groups. There were no differences in the cytokine mRNA and protein levels among the ascending colon, the descending colon, and the rectum of all groups.

**Conclusions:**

An increase in IFN-γ levels and a decrease in IL-10 levels were found in the intestinal mucosa of PI-IBS patients, suggesting that the infection may affect the Th1/Th2 balance. Thus, the dysregulation of the immune response is likely an important cause of IBS.

## Background

Irritable bowel syndrome (IBS) is a common intestinal disorder characterized by persistent or intermittent abdominal pain or discomfort, distention, and changes in stool patterns. Since IBS does not exhibit morphological or biochemical abnormalities, it has been viewed as a somatic manifestation of psychological stress. IBS is associated with abnormal intestinal motion and sensations, intestinal infection, hypothalamic-pituitary-gut axis dysregulation, and immune factors [1]. Several studies have found that 3% to 30% of patients develop IBS symptoms after intestinal infection [2], which is termed post-infectious IBS (PI-IBS). These symptoms predominantly arise from changes in intestinal mucosa permeability and motion that are induced by chronic immune disorders of the intestinal mucosa.

In 1986, Mosmann et al. categorized CD4+ T cells into Th1 and Th2 subgroups according to both cytokines secreted in long-term cultured murine cells and immune functions mediated. In recent years, many studies demonstrated that Th1 and Th2 play the different roles in mediation of immune responses [3]. Th1 cells predominantly secrete IFN-γ, IL-12, IL-2, and tumor necrosis factor-α (TNF-α), and mediate cellular immunity, while Th2 cells play a key role in promoting Th1 differentiation and the Th1 response. Th2 cells predominantly produce IL-4, IL-10, IL-13, and IL-6, and mediate humoral immunity. Studies confirm that Th1 and Th2 cells function as a pair of important regulators that restrain one another and produce cytokines, which interact to maintain a balanced immune response [4]. In a state of disequilibrium, the Th1/Th2 profile shifts and improper immunological response may induce IBS symptoms [5]. It has been confirmed that patients with both inflammatory bowel disease (IBD) and Th1/Th2 disequilibrium often develop IBS symptoms [6], which suggests that PI-IBS may result from abnormal Th1/Th2 immune regulation. Therefore, we chose the four main cytokines, IFN-γ, IL-12, IL-4 and IL-10, to observe the changes in Th1- and Th2-derived cytokines in the intestinal mucosa and to explore the Th1/Th2 shift and its potential role in PI-IBS patients.

## Methods

### Subjects and specimens

Thirty-eight IBS patients who had constipation-predominant type (C-IBS) or diarrhea-predominant type (D-IBS) according to the Roman III diagnostic criteria [7] were recruited for this study, including 20 PI-IBS patients with a history of acute enteritis and bacillary dysentery within the previous 3 to 12 months (13 men and 7 women, mean age: 49.71 ± 11.20 years) and 18 non-PI-IBS patients (8 men and 10 women, mean age: 40.52 ± 5.20 years). Twenty healthy people served as normal controls (11 men and 9 women, mean age: 43.74 ± 7.20 years) who had no intestinal symptoms, infections, immune rheumatic diseases, or history of anticoagulant administration. Mucosal specimens of each subject were collected from every region of the large intestine (ascending colon, descending colon, and rectum) with biopsy forceps (the same type was used for all sample collection); specimens were preserved in liquid nitrogen immediately for subsequent RNA extraction and protein assay. The study was carried out with institutional review board approval from Baogang Hospital, the third Affiliated Hospital of Inner Mongolia Medical College. All subjects provided written informed consent for endoscopy for study purposes.

### RT-PCR mRNA assay

Total RNA in the intestinal mucosa was extracted using Trizol solution (Invitrogen). The expression of cytokines, including IFN-γ, IL-12, IL-4, and IL-10 mRNA, were assayed by RT-PCR. The β-actin mRNA level was determined as an internal reference. Target genes and primers were listed in Table 1. The reverse-transcription was conducted at 70°C for 5 min, 37°C for 60 min, and 95°C for 5 min. The PCR cycling condition was 40 cycles at 50°C for 2 min, 95°C for 10 min, 95°C for 15 min, and 60°C for 1 min. The resultant products were resolved by electrophoresis. The gray values of the bands were calculated using Bandscan 4.5 software (Glyko). The relative mRNA expression levels of target genes were normalized to the corresponding internal standard.

**Table 1:** RT-PCR Primer sequences

**Cytokines**	**Primer sequence**
IFN-γ (90 bp)	Upstream 5′-GATGACTTCGAAAAGCTGACTAATTATTC-3′
	Downstream 5′-GTTCAGCCATCACACTTGGATGAG-3′
IL-12 (100 bp)	Upstream 5′-ATGATGGCCCTGTGCCTTAG-3′
	Downstream 5′-TGCC TCTTAGGATCCATCCATCAGAAG-3′
IL-4 (93 bp)	Upstream 5′-CACAAGCAGCTGATCCGATTC-3′
	Downstream 5′-TCTGGTTGGCTTCC TTCACAG-3′
IL-10 (123 bp)	Upstream 5′-CACAAGCAGCTGAT CCGATTC-3′
	Downstream 5′-TCTGGTTGGCTTCCTTCACAG-3′
β-actin (98 bp)	Upstream 5′-AGGTCATCACCATTGGCAATG-3′
	Downstream 5′-GGTAGTTTCGTGGATGCCACA-3′

### Western blotting for protein assay

The frozen specimens were thawed and then rinsed with PBS. Next, samples were lysed and fully homogenized in a buffer containing 15 mM NaCl, 1% NP-40, 0.1% SDS, 100 μM Aprotinin, 5 μM Leupeptin, 1 mM PMSF, 50 mM NaCl, at pH 7.3 and room temperature for 20 min. The protein concentration was determined and equal amounts of each sample were resolved electrophoretically on a polyacrylamide gel. The primary antibodies were IL-4, IL-10, IL-12 and IFN-γ (purchased from HuaAn Biotechnology Co., LTD, HangZhou). The β-actin protein was used as an internal standard. The western blot assay and coloration were conducted and the films were processed and scanned. The densities of the bands were assessed using image analysis software Bandscan 4.5. The relative protein levels of the target protein were obtained by correction with the corresponding internal standard.

### Statistical analysis

Statistical analysis was performed using SPSS 13.0 software. Data were expressed as the mean ± standard error (SE). One-way ANOVA tests were performed to assess the differences in the cytokine expression levels. A post-hoc Bonferroni test was used to assess the differences between the individual groups. P < 0.05 was considered significant.

## Results

### Cytokine mRNA levels in the intestinal mucosa

**Figure 1:**
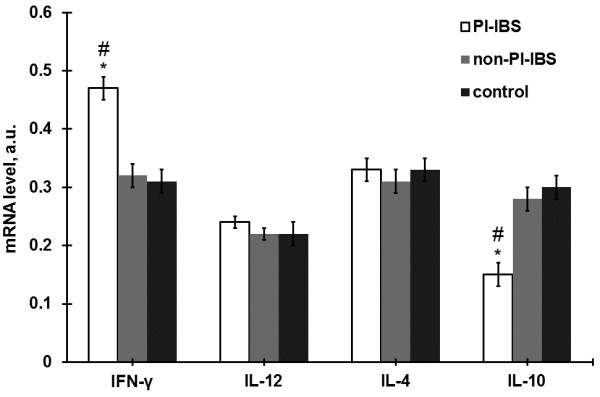
**Expression of the cytokine mRNA levels in the intestinal mucosa in the PI-IBS (n = 20), non-PI-INS (n = 18) and control groups (n = 20).** Data are presented as mean ± SD, *P < 0.05 versus controls, #p < 0.05 versus non-PI-IBS group.

**Figure 2:**
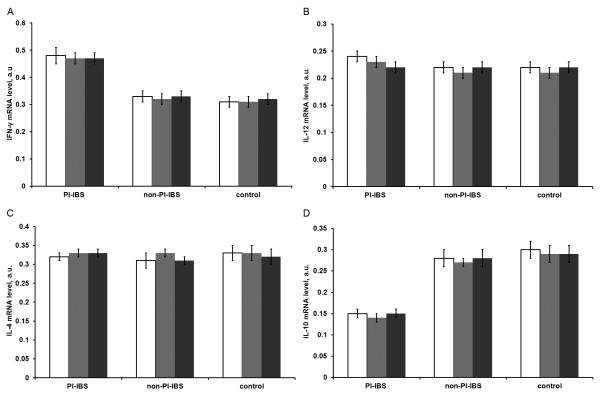
**Expression of the cytokine mRNA levels of: IFN-γ (A), IL-12 (B), IL-4 (C), and IL-10 (D) in different segments of the intestinal mucosa in the PI-IBS (n = 20), non-PI-INS (n = 18) and control groups (n = 20).** Blue: ascending colon, red: descending colon, green: rectum. Data are presented as mean ± SD. The cytokine mRNA expression levels showed no significant differences among the three segments within each group.

The results of RT-PCR assays showed that IFN-γ levels were significantly higher in the intestinal mucosa of PI-IBS patients (0.47 ± 0.02) than that in non-PI-IBS patients (0.32 ± 0.02) or healthy people (0.31 ± 0.02) (both P < 0.05), but there was no significant difference between the non-PI-IBS and control groups (P > 0.05). For IL-12 and IL-4 levels in the intestinal mucosa, no significant differences were noted in PI-IBS patients (0.24 ± 0.01 for IL-12 and 0.33 ± 0.02 for IL-4) compared to non-PI-IBS patients (0.22 ± 0.01 for IL-12 and 0.31 ± 0.02 for IL-4) or healthy people (0.22 ± 0.02 for IL-12 and 0.33 ± 0.02 for IL-4) (all P > 0.05), or between the non-PI-IBS and control groups (P > 0.05). Additionally, IL-10 mRNA level was significantly lower in the intestinal mucosa of PI-IBS patients (0.15 ± 0.02) than in non-PI-IBS patients (0.28 ± 0.02) or healthy people (0.30 ± 0.02) (both P < 0.05), but there was no significant differences between the non-PI-IBS and control groups (P > 0.05; Figure 1). Furthermore, IFN-γ, IL-12, IL-4, and IL-10 mRNA levels showed no significant differences among the ascending colon, descending colon, and rectal mucosa within each group (all P > 0.05; Figure 2).

### Proteins of various cytokines in the intestinal mucosa

**Figure 3:**
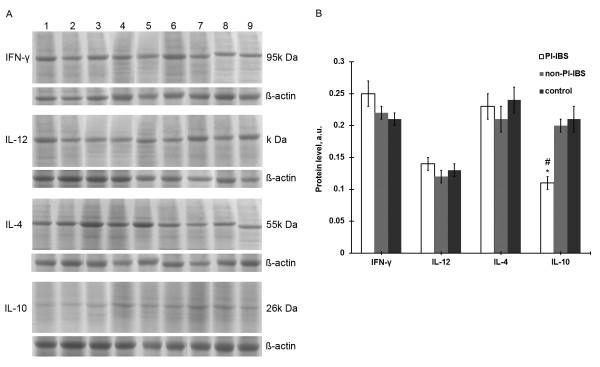
**The cytokine protein expression levels in the intestinal mucosa in the PI-IBS (n = 20), non-PI-INS (n = 18) and control groups (n = 20).** A. Representative graphs of the Western blot assay. Lane 1–3 PI-IBS; Lane 4–6 non-PI-IBS; Lane 7–9 healthy control group. B. Quantitative of expression of cytokines. Data are presented as mean ± SD. *P < 0.05 versus controls, #p < 0.05 versus non-PI-IBS group.

**Figure 4:**
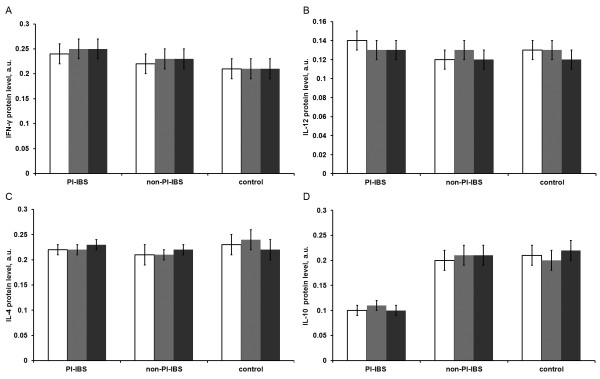
**Cytokine protein expression levels of IFN-γ (A), IL-12 (B), IL-4 (C), and IL-10 (D) in the intestinal mucosa of the different segments of the PI-IBS (n = 20), non-PI-INS (n = 18) and control groups (n = 20).** Blue: ascending colon, red: descending colon, green: rectum. Data are presented as mean ± SD. The cytokine protein levels showed no significant differences among the three segments within each group.

IFN-γ, IL-12, IL-4 and IL-10 protein expression in the intestinal mucosa was further measured by western blotting. As shown in Figure 3, the IFN-γ protein level showed a trend toward up-regulation in PI-IBS patients (0.25 ± 0.02) but not in non-PI-IBS patients (0.22 ± 0.01) or healthy people (0.21 ± 0.01); however, the differences were not significant (both P > 0.05). There was also no significant difference between non-PI-IBS and control groups (P > 0.05). Also, there were no significant differences between the levels of IL-12 and IL-4 in the intestinal mucosa in the PI-IBS (0.14 ± 0.01 of IL-12 and 0.23 ± 0.02 of IL-4), non-PI-IBS (0.12 ± 0.01 of IL-12 and 0.21 ± 0.02 of IL-4), and control groups (0.13 ± 0.01 of IL-12 and 0.24 ± 0.02 of IL-4) (both P > 0.05). In addition, IL-10 expression was significantly lower in PI-IBS patients (0.11 ± 0.01) compared to non-PI-IBS patients (0.20 ± 0.01) or healthy people (0.21 ± 0.02) (both P < 0.05). Moreover, no significant differences were detected in IFN-γ, IL-12, IL-4, and IL-10 expression among the ascending colon, descending colon, and rectal mucosa in each group (all P > 0.05; Figure 4).

## Discussion

Emerging evidence revealed that intestinal infection and inflammation play a crucial role in the occurrence and development of IBS [8,9]. Previous study above 70% D-IBS and 10% C-IBS were involved in infection [10]. In the present study, we showed that 20 of 38 (above 52%) D-IBS and C-IBS patients had an intestinal infection and bacillary dysentery, which confirmed the role of infection in the disease. Moreover, we found that the expression of cytokines was imbalanced and shifted between T helper 1 and T helper 2 (Th1/Th2) in intestinal mucosa of PI-IBS patients, suggesting that an immune dysregulation mechanism was involved in the disease.

CD4^+^T cells can be categorized into Th1 and Th2 subgroups according to the differences in cytokine secretion, and the two subgroups restrain and transform mutually to maintain normal immunological function [11]. Several studies suggested that Th1-derived cytokines IFN-γ and IL-2 in peripheral blood were significantly higher in D-IBS patients than that in healthy persons, while the Th2-derived cytokine IL-4 was significantly lower in D-IBS patients than in healthy persons [1], illustrating that the changes in immune balance were primarily related to decreasing cytokine secretion [2]. In this study, our results showed that compared to non-PI-IBS patients or healthy people, Th1-derived cytokine IFN-γ expression was significantly upregulated in the intestinal mucosa of PI-IBS patients. In addition, the expression level of the Th2-derived cytokine IL-10 was significantly lower in the intestinal mucosa of PI-IBS patients. However, the expression of IFN-γ or IL-10 between the non-PI-IBS and control groups was not significantly different. These results suggested that expression of Th1-derived cytokines increased while Th2-derived cytokines decreased in PI-IBS patients, which implied that the Th1/Th2 balance was possibly shifted to Th1 immunodominance. Therefore, it is indicated that the Th1/Th2 shift would be affected by the infection. In the infectious state, the inflammatory factors would change the intestinal mucosal permeability and the subsequent immunocyte response, such as eliciting the Th1 response by microbiotic antigens. In addition, this Th1/Th2 shift may lead to sustained immunological activation and subsequent abnormal sensation through the neuro-endocrine-immunity network [12]. However, further studies are still required to clarify the exact mechanism.

IFN-γ and IL-12 are proinflammatory factors. IFN-γ can combine with specific receptors to exert distinct effects, such as macrophage activation, promotion of Th0 cells to differentiate into Th1 cells, inhibition of cell proliferation, promotion of B cell differentiation, and antibody production. In contrast, IL-4 and IL-10 are produced by Th2 cells and are important immune regulators and anti-inflammatory factors; they inhibit Th1 cell proliferation, promote B cell proliferation, antagonize the response to antigenic stimulation, block harmful immunological responses, depress the function of mononuclear macrophages and granulocytes, and downregulate mononuclear macrophage-secreting proinflammatory factors such as IL-1β and TNF-α. IL-4 and IL-10 may decrease the mucosal inflammation that arises from Th1 cytokines. Previous studies have shown an increase in inflammatory cells, proinflammatory factors, and immunological activation in the intestinal mucosa of PI-IBS patients [13,14]. In this study, IFN-γ expression increased and IL-10 expression decreased in the intestinal mucosa of PI-IBS patients, which implies that persistent inflammation may exist due to dysregulation of the these cytokines. Since the permeability changes in the intestinal mucosa are caused by inflammatory infections, the immunological cells’ contact with certain foods and microorganisms would easily cause activation, and lead to Th1 immunodominance. The increase of cellular immunity and the action of the secreted cytokines may result in abnormal intestinal function, motion, and a series of symptoms [15,16]. These results are also consistent with PI-IBS symptoms [17,18].

Some studies indicate that Th1- and Th2-derived cytokines show different expression levels in different segments of the intestine in several IBS patients and the quantity of immunocytes varies with the intestinal segments [19,20]. We chose the ascending colon, the descending colon, and the rectum to observe whether there is variability in the expression Th1- and Th2-derived cytokines in the different locations. We did not see any significant difference in the Th1- and Th2-derived cytokines expression level in the three intestinal segments. However, a larger sample size is needed to clarify these results.

## Conclusions

In conclusion, this study showed that there was a Th1/Th2 immunological shift in the intestinal mucosa of PI-IBS patients. The occurrence and development of PI-IBS may strongly correlate with intestinal inflammation and changes of inflammatory factors. Our results provide a theoretical basis for exploring PI-IBS treatment.

## Abbreviations

C-IBS, Constipation-predominant type; D-IBS, Diarrhea-predominant type; IBD, Irritable bowel disease; IBS, Irritable bowel syndrome; IFN-γ, Interferon-gamma; IL-1β, Interleukin-1beta; IL-4, Interleukin-4; IL-6, Interleukin-6; IL-10, Interleukin-10; IL-12, Interleukin-12; PI-IBS, Post infectious irritable bowel syndrome; Th1, T helper 1; Th2, T helper 2; TNF-α, Tumor necrosis factor-alpha.

## Competing interests

The authors declare that they have no competing interests.

## Authors’ contributions

This topic was designed by YZ and ZD; JC carried out molecular genetic studies, the research process, data analysis and writing paper. All authors read and approved the final manuscript.

## Pre-publication history

The pre-publication history for this paper can be accessed here:

http://www.biomedcentral.com/1471-230X/12/91/prepub
